# Virchow and Koch

**Published:** 1891-01-24

**Authors:** 


The Hospital

A Weekly Journal of

Science, Medicine, Nursing, and Philanthropy.

*For Hospitals, Asylums, Infirmaries, Dispensaries, Medical Practitioners, Students, Nurses, and the Charitable Public.*

Vol. IX.—No. 226.] [January 24, 1891.

### Virchow and Koch.

It is becoming clearer as experience increases that Koch's remedy is not a cure for pronounced cases of consumption. So far as present observation extends, it would seem that other known methods of treating phthisis give surer hope of good results than that of Koch, whilst they have the merit of being entirely free from the risks and dangers which attend his treatment. It is possible that a definite use may be found in medicine for the new lymph, though whether it will continue to be used for any length of time as a remedy for consumption no wise man would at present venture to predict. What medical men have now to do is to deal with the substance in an experimental way, without any regard to Koch's conclusions, and as if the lymph were possessed of absolutely unknown properties, and had been discovered by each individual experimenter for himself. Above all things, they must weigh the testimony of witnesses, and also the witnesses themselves, as well as count heads.

One fact of a very instructive character has emerged during the progress of the "Koch lymph" history. It is this: that scientific persons, even when considering and discussing scientific questions, are in all respects exceedingly like ordinary mortals. They follow leaders; they take sides; they rush to conclusions with unscientific haste; they make partial observations; they believe what they wish to believe rather than what the facts testify; and, having once taken up a position, they can hardly be compelled to move an inch, even at the bayonet's point of absolute and unquestionable demonstration.

A crisis like that through which we have just been passing acts as a differentiating test upon men of established scientific status. It adds to the reputation of some; it diminishes or destroys that of others. It shows how much of the truly scientific temper each man possesses. Has he a large and open mind? Is he candid and impartial? Can he consider facts apart from persons? Dare he stand by himself against a flood-tide of scientific excitement? Can he discriminate between the essential and the accidental? And will he speak as he finds, though the heavens should fall? Tried by these tests, some men of science, both in this country and on the Continent, have emphatically been found wanting. They must bear in mind, however, that these are exactly the kinds of tests which will be applied to their reputations when the sum of each life is added up; and according as they are able or otherwise to stand such testings will their fame be permanently enduring, or evanescent as the dew. As in literature, so in science—the man whom his contemporaries thought to be a giant may be proved by posterity to have been a pigmy.

Professor Rudolph Virchow is one of those men who seem designed by nature to stand steadfast in the midst of storms. He has not, so far as we are aware, made any pronouncement of an authoritative kind on the Koch crisis until quite recently. Speaking at the Berlin Medical Society, in the discussion on Professor B. Fraenkel's paper, he said: "In the first place, I wish to say that I do not, as will be readily understood, propose to speak here of my own casual observations on patients, but only of what we have been able to establish by way of anatomo-pathological research. From the beginning of the injection period up to the end of the year that has just passed away, the total number of deaths of patients treated by injections of Koch's fluid that have come before us has been twenty-one. Besides these we have already had, in the course of the present year I believe, six or seven other cases. . . . . In addition to this, my assistants have made necropsies in a large number of similar cases in other hospitals, and in private, and I have seen the most important results of these examinations." Now, in the first place, it is clear that a very large number of persons have died after injections of Koch's lymph. Because in such a short time as two months one pathologist alone has had opportunities of examining no fewer than twenty-six dead persons who had been treated; and his assistants have examined what he calls a "large number" of similar cases. Koch's cure is said to have a particular effect upon lupus, and to cure it in a particular way; and it is assumed that it has the same or a similar effect upon lung tubercle, and to cure it in a similar way. Of course, in cases that do not die no one can tell in what way the tubercle in the lung may have been affected, because there is no means of exposing the cured lung to view. But in cases that die the lungs can be examined both by the naked eye and by the microscope: and it can be seen quite easily whether the effect upon tubercles in the lung or other internal organ is similar to that upon lupus in the skin, or whether there is a different effect: whether the effect, whatever it may be, is beneficial or injurious: or whether there be no perceptible effect at all. It is clear, in short, that in this particular case, where the remedy under discussion is stated to have such an exceedingly obvious effect upon external tissues, and where its cure of an internal malady is said to depend upon its similar effect upon similar internal tissues,—it is clear, we say, that in such a case as this the evidence of the post-mortem-table should be proof positive for or against the value of the remedy in internal tuberculosis.

What, then, was the state of the internal tubercular tissues in many of the cases examined by Professor Virchow in the dead-house? Did it show that a reaction had been set up at the tubercular points alone, and that every tubercular focus had been reduced to a necrotic condition? It did not show anything of the kind. Acute hyperæmias were set up; but these were not confined to the tubercular foci by any means. On the contrary, in a case of tubercular arachnitis, not only was there a general engorgement of the vessels of the pia mater, but also a dusky-red appearance of the unaffected brain substance beneath. But what is of still greater importance is the fact that though the arachnoid membrane and the deeper brain substance in the case examined were in such a condition of extreme hyperæmia, the actual tubercular tissue does not seem to have been affected at all. Says Professor Virchow: "I personally examined the tubercles; I cannot, however, say that I saw any evidence in them of retrogressive changes. The tubercles were very well formed and presented the usual appearances of meningeal tubercles."

Now what is the significance of this fact? It shows that the Koch lymph may not only fail to cure tubercle, but that it may also fail to detect it, or even to touch it in any way. It shows, in short, that the lymph may be just as useless for diagnostic purposes as it may be for purposes of cure. Effects such as have been described were not confined to those cases in which the tubercular disease primarily affected the brain. To quote Professor Virchow again: "Similar acute hyperæmias and swellings are also seen in other internal parts. In particular, it was repeatedly noted by us that even the surface of old pulmonary cavities showed unusually intense redness of the granulation layers; moreover, hæmorrhagic infiltrations of walls were not seldom present, and even recent hæmorrhages were observed in the cavities. The processes . . . are not merely transient congestive swellings. . . . But there can be no doubt that in the internal parts actual inflammatory processes and especially active proliferations, occured to an intense degree. . . . The lymphatic glands presented a quite unusual degree of enlargement, and notably that form of medullary swelling characteristic of acute irritations, which is caused by rapid proliferation of the cells in the interior of the glands. It is in harmony with these large acute swellings that frequently also an increase in the colourless elements of the blood can be detected—a condition of leucocytosis. . . . These swellings occasionally assume a very dangerous character."

So far we have considered but a small portion of Professor Virchow's judicial and admirably scientific paper. But we have quoted enough to justify two assertions: first, that our original contention set forth in The Hospital immediately after the appearance of Koch's first paper, that the reaction caused by the injection of the lymph is not a reaction exclusively confined to tubercular tissue, but is, to a large extent, a general reaction, in which the tubercular tissues have no more than a share, is being confirmed; and, secondly, that so far from the reaction being a tubercular reaction only, we may have a general reaction, so pronounced in particular parts as to kill the patient, in which, notwithstanding, the tubercular foci shall not be so much as touched. The paper of Professor Virchow must give every medical man pause who has the remotest claim to the understanding of elementary pathological facts. The position which we have reached in the history of the Koch lymph is this, and no other: that a powerful fluid has been manufactured; and that it remains for sober practitioners to prove by a sufficient induction, whether or not it will ever be of any value to mankind.

